# Short- and long-term outcomes in infective endocarditis patients: a systematic review and meta-analysis

**DOI:** 10.1186/s12872-017-0729-5

**Published:** 2017-12-12

**Authors:** Tadesse Melaku Abegaz, Akshaya Srikanth Bahagavathula, Eyob Alemayehu Gebreyohannes, Alemayehu B. Mekonnen, Tamrat Befekadu Abebe

**Affiliations:** 1 Department of Clinical pharmacy, School of Phamacy, College of Medicine and Health Sciences, University of Gondar, Gondar, Ethiopia; 2 Medication Safety Chair, College of Pharmacy, King Saud University, Riadh, Saudi Arabia; 3 Master’s Program in Health Economics, Policy and Managment; Student; Department of Learning, Informatics, Managent and Ethics, Karolinska Institutet, Solna, Sweden

**Keywords:** Infective endocarditis, Long-term mortality, Meta-analysis, Short-term mortality

## Abstract

**Background:**

Despite advances in medical knowledge, technology and antimicrobial therapy, infective endocarditis (IE) is still associated with devastating outcomes. No reviews have yet assessed the outcomes of IE patients undergoing short- and long-term outcome evaluation, such as all-cause mortality and IE-related complications. We conducted a systematic review and meta-analysis to examine the short- and long-term mortality, as well as IE-related complications in patients with definite IE.

**Methods:**

A computerized systematic literature search was carried out in PubMed, Scopus and Google Scholar from 2000 to August, 2016. Included studies were published studies in English that assessed short-and long-term mortality for adult IE patients. Pooled estimations with 95% confidence interval (CI) were calculated with DerSimonian-Laird (DL) random-effects model. Sensitivity and subgroup analyses were also performed. Publication bias was evaluated using inspection of funnel plots and statistical tests.

**Results:**

Twenty five observational studies (retrospective, 14; prospective, 11) including 22,382 patients were identified. The overall pooled mortality estimates for IE patients who underwent short- and long-term follow-up were 20% (95% CI: 18.0–23.0, *P* < 0.01) and 37% (95% CI: 27.0–48.0, P < 0.01), respectively. The pooled prevalence of cardiac complications in patients with IE was found to be 39% (95%CI: 32.0–46.0) while septic embolism and renal complications accounted for 25% (95% CI: 20.0–31) and 19% (95% CI: 14.0–25.0) (all P < 0.01), respectively.

**Conclusion:**

Irrespective of the follow-up period, a significantly higher mortality rate was reported in IE patients, and the burden of IE-related complications were immense. Further research is needed to assess the determinants of overall mortality in IE patients, as well as well-designed observational studies to conform our results.

**Electronic supplementary material:**

The online version of this article (10.1186/s12872-017-0729-5) contains supplementary material, which is available to authorized users.

## Background

Infective endocarditis (IE) is an infection of the endocardial lining of the heart with pre-existing lesions or on intra-cardiac foreign materials [1]. Bacterial species such as staphylococcus and streptococcus accounts for 80% of cases; however, it may occasionally be due to fungal pathogens as well [1, 2]. The Global Burden of Diseases Study—GBD 2010 reported a crude IE incidence ranged between 1.5 to 11.6 cases per 100,000 people and the mean proportion of patients that underwent valve surgery was 32.4 ± 18.8%, and the mean fatality risk was 21.1 ± 10.4% [3]. The presence of rheumatic heart disease, congenital heart disease, prosthetic valves and previous episodes of IE are some of the traditional risk factors; however, predisposing factors such as intra-cardiac devices, intravenous drug use, human immune virus (HIV) infection, diabetes, hemodialysis, degenerative valvular heart disease and dental infection are some of the risk factors that predominates over the traditional risk factors [2].

Clinical suspicion of IE is very often delayed because early clinical symptoms are not properly evaluated and present as a subacute disease with symptoms like fever and malaise that does not correspond to a serious disease. Blood culture-positive endocarditis (BCPE) and blood culture-negative endocarditis (BCNE) remain the cornerstone of diagnosis and provide significant array for identification and susceptibility testing. IE can mimic many diseases and prompt diagnosis remains a challenge with high in-hospital morbidity and mortality, and compromised short-term outcomes after hospital discharge [4]. Delay in antibiotic therapy and inappropriate antibiotics in suspected IE cases has negative effects on clinical outcomes in acute stage [5]. Successful microbial eradication by antimicrobial drugs or by surgical removal of infected materials and draining abscesses are essential for positive outcomes.

Despite advances in medical knowledge, technology and antimicrobial therapy, IE is still associated with devastating outcomes and becoming a pressing problem, with at least one in four died of IE [3]. The in-hospital mortality (22%) and 5-year mortality (45%) was significantly higher in IE cases, with an annual deaths of 48,300 patients globally in 2010 [6]. In fact, several discrepancies have been noticed in the literature concerning the impact of guidelines, recommendations, risk estimations and research findings estimating the outcomes in IE [7–11]. Several reviews were focused examining the effect of different antibiotic regimens [12], optimal timing of surgery [13], epidemiology [14] and effect of surgical intervention [15]. However, no reviews have yet assessed the outcomes of IE patients undergoing short-term and long-term treatment, heart valve involvement and outcomes in intravenous drug users. We, therefore, conducted a systematic review and meta-analysis including a wide variety of studies examining the short-term and long-term outcomes in IE patients. The main outcome measures were clinical outcomes and overall mortality.

## Methods

### Data sources and search strategy

A computerized systematic literature search was carried out using the scientific databases: PubMed, Scopus and Google Scholar. We exhaustively searched the databases for studies published between 2000 to August, 2016 using the following key words: ‘infective endocarditis’ in conjunction with search terms such as ‘long- or short-term outcome’, ‘prognosis’, ‘in-hospital’, ‘mortality’, ‘native valve’, ‘prosthetic valve’, and ‘drug users’.

### Study selection and eligibility

#### Study selection

All records that were identified from searches of the electronic databases were loaded into the ENDNOTE software version X5 (Thomson Reuters, USA) and duplicates were removed. Two author (TMA and EAG) screened the titles and abstract of each reference identified by applying the inclusion criteria. Two authors (TMA and TBA) independently collected the full-text and reviewed them. Final inclusion of the studies was determined by agreement of both reviewers and involvement of the third author (EAG) in case of discrepancy. All the authors involved in the discussion and agreed on the final inclusion.

#### Inclusion and exclusion criteria

Literature reviews and studies with only surgical intervention were excluded. But, if both medical and surgical interventions were undertaken for a patient, the study was included. Studies that did not determine the short- or long-term outcome were excluded. For outcome evaluation, patients with definite IE and patients who fulfilled the modified Duke criteria for diagnosis of IE, were considered. We also included studies that assessed IE in Intravenous (IV) drug users and those with prosthetic device. Age was limited to adults and our search term did not include children or pediatrics. Only studies published in English were considered eligible. In addition, studies with small samples size (less than 50) were excluded to maintain the quality of our findings.

### Data extraction and quality assessment

Data on socio-demographic characteristics including age, sex, study design, study setting, and mean follow-up period were retrieved. Clinical profiles of patients such as the type of valve affected, bacterial profile and primary outcomes including short-and long-term mortality were extracted. Mortality is included as all-cause mortality. Mortality within 30 days of admission and in-hospital mortality was classified as ‘short-term’ mortality but if both in-hospital mortality and the 30 day mortality was given, the latter was chosen. Whereas, long-term mortality denotes mortality after patients have been discharged from the hospital and died after 30 days of follow-up. For ease of analysis, studies that reported the long-term outcome at various time intervals, we employed the longer duration of time in the analysis. Also, secondary outcomes were collated and defined in this study as complications due to IE such as cardiac damage, renal failure and embolic complications.

The quality of the studies was evaluated using STROBE (Strengthening the Reporting of Observational Studies in Epidemiology) scale [16]. Accordingly, we arbitrarily classified included studies into high quality (≥75% of the STROBE checklist) and low quality (<75% of the STROBE checklist).

### Statistical analysis

The meta-analysis was carried out with OpenMetaAnalyst (http://www.cebm.brown.edu/openmeta) and publication bias was assessed using Comprehensive Meta-analysis version-3 (Biostat, Englewood, New Jersey, USA). The random effects model was used for combining results of included studies in the meta-analysis. The heterogeneity in pooled estimation was determined by the DerSimonian-Laird (DL) approach and was assessed using I^2^. Sensitivity and subgroup analyses were conducted to determine the robustness of the results and sources of variation in pooled estimation, respectively. Initially, we planned to stratify primary and secondary outcomes on the basis of various sources of variation such as sex, age, and types of IE. However, included studies did not provide these data in extractable form and/or because there were inconsistencies in reporting the subgroups, we did not able to conduct subgroup analysis for our primary and secondary outcomes. On post hoc *analysis*, rather we conducted subgroup analysis based on the incidence and prevalence of IEs, stratified according to sex (male vs. female) and types of IE (native, prosthetic and drug users). Moreover, publication bias for the primary outcome was assessed by Egger and Begg’s tests and inspection of funnel plots.

## Results

**Fig. 1:**
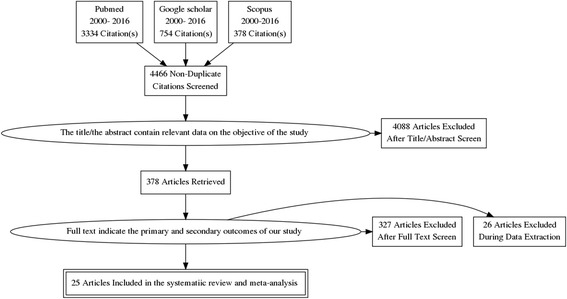
Flow chart indicating the selection process of studies

A total of 4466 unique articles were identified from three databases: PubMed (3334), Scopus (378) and Google Scholar (754), of which fifty-one deemed eligible for the full-text review and twenty-five articles were finally included in the systematic review and meta-analysis (Fig. 1).

### Characteristics of included studies

Overall, 15 included studies were from Europe [], six from Asia [32 33 34 35 36 37], one from Africa [38], and the remainder studies were intercontinental encompassing many countries [39 40 41]. We identified 14 retrospective and 11 prospective studies. The sample sizes in the included studies ranged from 66 (minimum) [34] to 8494 (maximum) [33]. A total number of 22,382 patients were included in the review. The mean age of the study subjects in the studies ranged from nearly 23 to 80 years of age [17 23], with 42% to 80% males [21 32]. All but one study [27] reported short-term outcome. Whereas, 14 studies estimated long-term outcome [17 18 19 20 22 23 24 25 26 27 28 29 30 39]. Three studies [22 27 29] estimated ten year outcome and six studies [17 23 24 25 28 29] assessed five year outcome, and the remainder studies [18 22 25 26 27 39] evaluated this outcome at one year. Secondary outcomes (complications of IE) were also evaluated in 17/25 of the included studies. The methodological qualities of included studies were variable and there was none that met the complete STROBE criteria (Table 1).

**Table 1:** Overview of studies included in the systematic review and meta-analysis

Study	Study design	Location	Sample size	Mean age, years	Sex, % (F:M)	Outcomes (primary and secondary)					% STROBE criteria met
						Short-term mortality	Long-term mortality	Renal Complications	Cardiac complication	Embolic complications	
Ternhag et al. 2013 [17]	Prospective	Sweden	7603	65.7	41:59	√	√	–	–	–	86
Martinez-Sellés et al. 2008 [18]	Prospective	Spain	222	63.5 ± 15.5	36:64	√	√	–	–	–	73
Fernandez-Hidalgo et al. 2012 [19]	Prospective	Spain	438	–	35:65	√	√	–	–	–	91
Samol et al. 2015 [20]	Retrospective	Germany	216	62 ± 14	31:69	√	√	√	√	√	82
Pazdernik et al. 2016 [21]	Retrospective	Czech Republic	106	57 ± 14.8	20:80	√	–	√	√	–	82
Thuny et al. 2008 [22]	Prospective	France	95	53 ± 16	27:73	√	√	–	√	√	82
Remadi et al. 2009 [23]	Prospective	France	348	79.8 ± 4	28:72	√	√	√	√	√	91
Krecki et al. 2007 [24]	Retrospective	Poland	69	52 ± 12	41:59	√	√	√	√	–	77
Moreno et al. 2002 [25]	Prospective	Spain	151	66 ± 11 versus 50 ± 19 years	34:66	√	√	√	√	√	78
Tran et al. 2006 [26]	Retrospective	Denmark	132	54 (range:19–83)	37:63	√	√	–	–	–	64
Mirabel et al. 2014 [27]	Prospective	France	198	61.1 (range:15.5–71)	30:70	–	√	√	√	–	95
Ferreira et al. 2013 [28]	Prospective	Portugal	147	63 ± 11	29:71	√	√	–	√	√	82
Leroy et al. 2015 [29]	Retrospective	France	248	62.4 ± 13.3	36:64	√	√	√	√	√	77
Netzer et al. 2002 [30]	Retrospective	Europe	212	53.6 ± 13.9	25:75	√	√	√	√	√	86
Wallace et al. 2002 [31]	Retrospective	United Kingdom	208	52 ± 1.2	34:66	√	–	–	–	–	68
Khaled et al. 2010 [32]	Prospective	Yemen	72	28.6 ± 14.5	58:42	√	–	√	√	√	86
Shih et al. 2014 [33]	Population based cohort study	Taiwan	8494	56.2 ± 19.2	36:64	√	–	√	√	√	82
Tariq et al. 2004 [34]	Retrospective	Pakistan	66	28.6 ± 12.3	33:67	√	–	–	–	–	64
Tariq et al. [35]	Retrospective	Pakistan	159	34.6 ± 20.7	35:65	√	–	–	–	–	76
Garg et al. 2005 [36]	Retrospective	India	192	27.6 ± 12.7	27:73	√	–	√	√	√	73
Math et al. 2010 [37]	Prospective	India	104	23.3 ± 9.56	29:71	√	–	√	√	√	73
Letaief et al. 2007 [38]	Retrospective	Tunisia	435	32.4 ± 16.8	44:56	√	–	–	√	√	77
Athan et al. 2012 [39]	Prospective	Multicounty	177	–	26:74	√	√	–	–	–	79
Lauridsen et al. 2015 [40]	Prospective	Multicounty	727	–	31:69	√	–	–	√	√	95
Lalani et al. 2010 [41]	Prospective	Multicounty	1552	57	31:69	√	–	–	√	√	95

√- denotes inclusion in the respective studies; (−) refers ‘not stated’

### Clinical characteristics and risk factors

A total of 1974 (8.8%) patients were having IE due to mitral valve infection while 2162 (9.7%) were due to aortic valve involvement, and combination of valves were reported in 18,246 (81.5%) patients. While 2278 (10.2%) patients had acquired left sided IE, the remaining constituted both left and right sided IE, 20,104 (89.8%). Besides medical intervention, only 3496 (15.6%) patients underwent surgical intervention.

Data from 10,987 patients were available to determine the types of IE. Of these, 8496 (77.3%) had native valve IE and prosthetic valve IE was identified in 1414 (12.9%) patients. Whereas, IE due to intravenous drug use was reported in 1077 (9.8%) subjects. Other patients, 11,395 (of the 22,382) had either mixed type or unclassified IE.

A total of 5011 (22.4%) patients were identified to have risk factors including congenital heart disease (CHD) which was reported as a predisposing factor in 220 patients, whereas rheumatic heart disease (RHD) was reported in 513 cases. About 63 patients experienced previous episodes of IE.

### Common pathogens involved in IE

Most of the studies did not report the number of species involved in causing IEs. Among the studies that reported culture results, it was found that 63% (1320/2012) cases were positive and negative in 21% (1049/12,508) subjects. Culture was not performed or adequately documented in the rest of individuals. Among the reported pathogens, the dominant strain was *Staphylococcus aureus* (2894/13,768; 27%) followed by *Streptococcus pneumonia* (2426/13,768; 23%) (Table 2).

**Table 2:** Common pathogens involved in the pathogenesis of IEs

Pathogens/culture	Patients with pathogens	Total number of patients	Overall estimate, 95% CI	References
Culture positive	1320	2012	0.63(0.37–0.88)	[22, 24–26, 28, 29, 31, 32, 35–38]
Culture negative	1049	12,508	0.21(0.09–0.42)	[21, 22, 24–26, 31, 33, 35, 36, 38–41]
*Staphylococcus aureus*	2894	13,768	0.27(0.22–0.33)	[19, 21, 22, 24–33, 36–41]
Streptococcus aureus	2426	13,768	0.23(0.18–0.29)	[19, 21, 22, 24–33, 36–41]
Enterococci bacteria	313	2731	0.11(0.10–0.28)	[19, 21, 22, 24–26, 39, 41]
HACEK and others	628	11,936	0.10(0.10–0.11)	[19, 21, 22, 24–29, 31, 33, 36–40]

*Abbreviation: HACEK* Haemophilus, Aggregatibacter, Cardiobacterium hominis, Eikenella corrodens, Kingella species

### Study outcomes

#### Primary outcomes

**Fig. 2:**
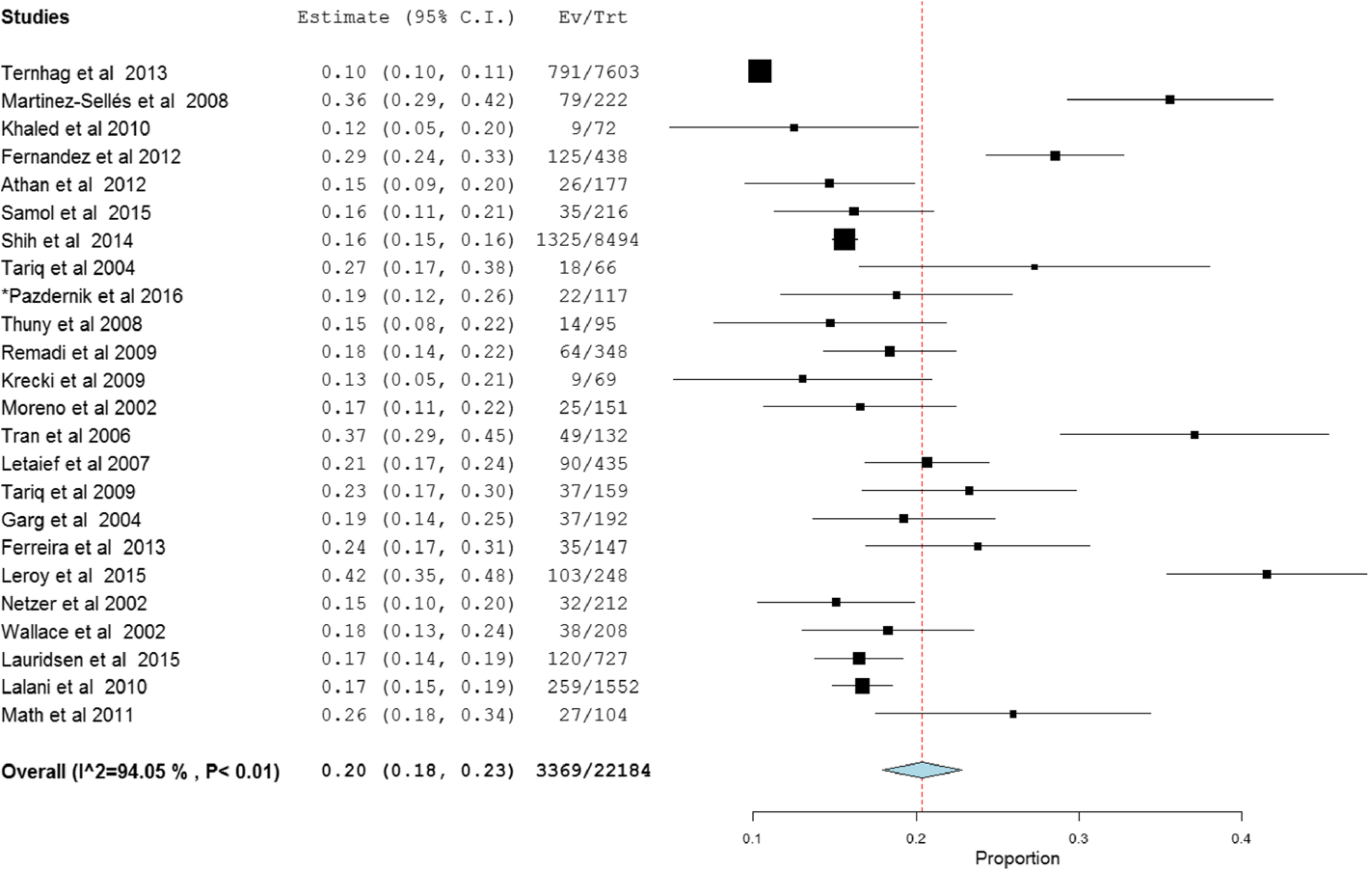
Short-term outcome of infective endocarditis. *117 episodes of care for 106 patients were occurred and 117 was used as a denominator in the calculation

**Fig. 3:**
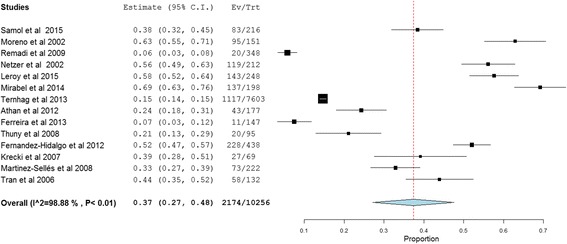
Long-term outcome of infective endocarditis

In total, the population for the assessment of mortality consisted of 22,382 subjects for both long- and short-term outcomes. Short-term outcome was determined by analyzing the data of 22,184 patients obtained from 24 studies, whereas long-term mortality was analyzed using the 10,256 patients included from 14 studies. Short-term mortality occurred in 3369 patients while long-term follow-up resulted in death of 2174 patients. The overall pooled mortality estimates for IE patients who underwent short- and long-term follow-up were 20% (95% CI: 18.0–23.0, *P* < 0.01; heterogeneity I^2^ = 94.0%) and 37% (95% CI: 27.0–48.0, *P* < 0.01; heterogeneity I^2^ = 98.9%), respectively (Figs. 2 and 3).

#### Secondary outcomes

**Fig. 4:**
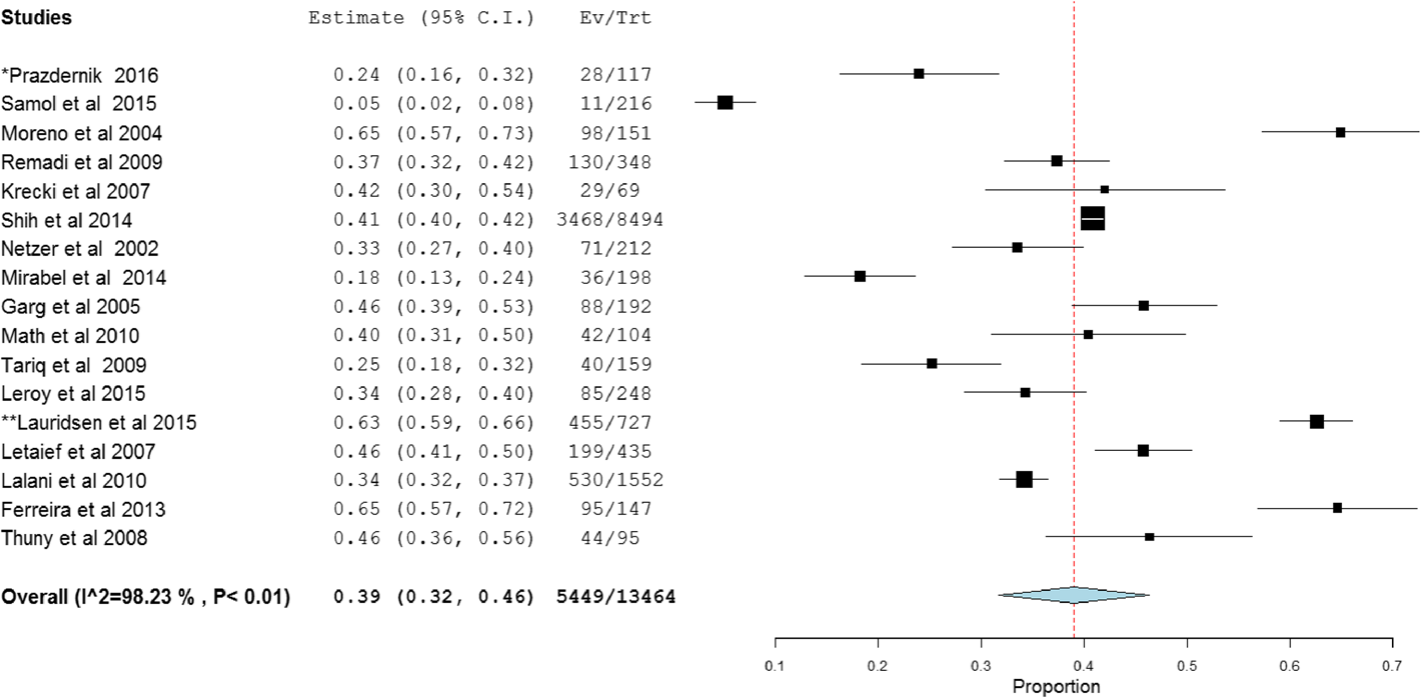
Cardiac complications of infective endocarditis. *117 episodes of care for 106 patients were occurred and 117 was used as a denominator in the calculation. **Data for cardiac complication extracted from other presented data

**Fig. 5:**
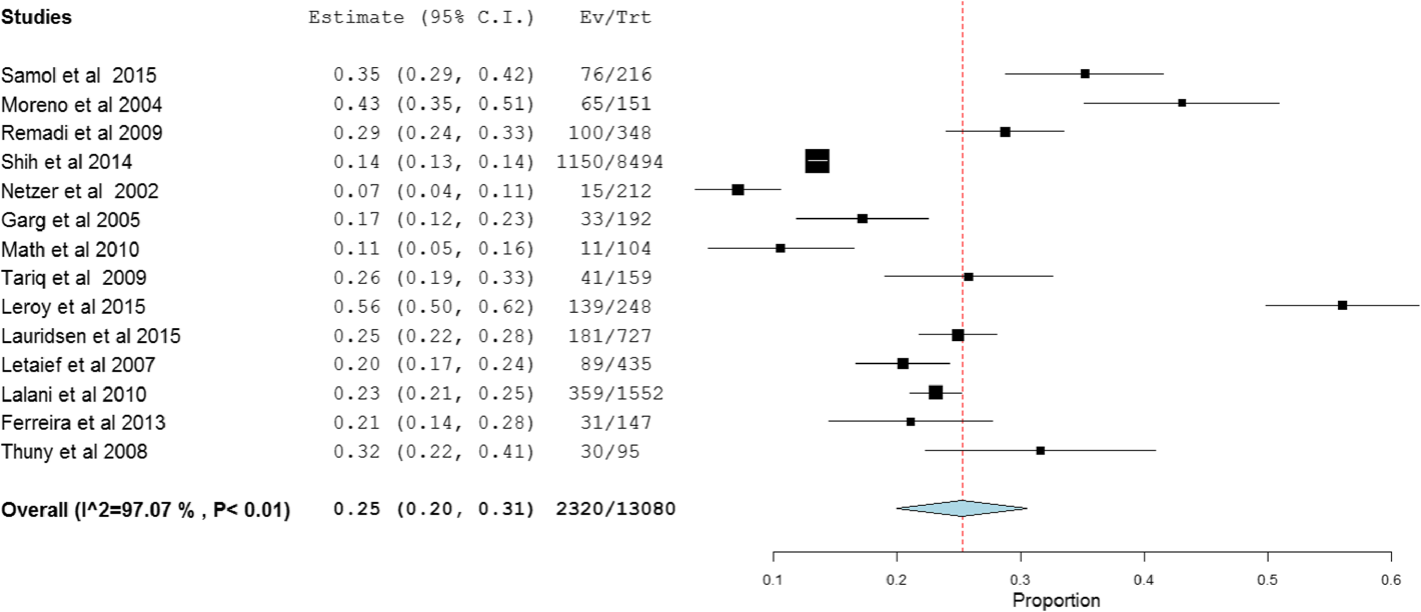
Embolic/septic complications of infective endocarditis

**Fig. 6:**
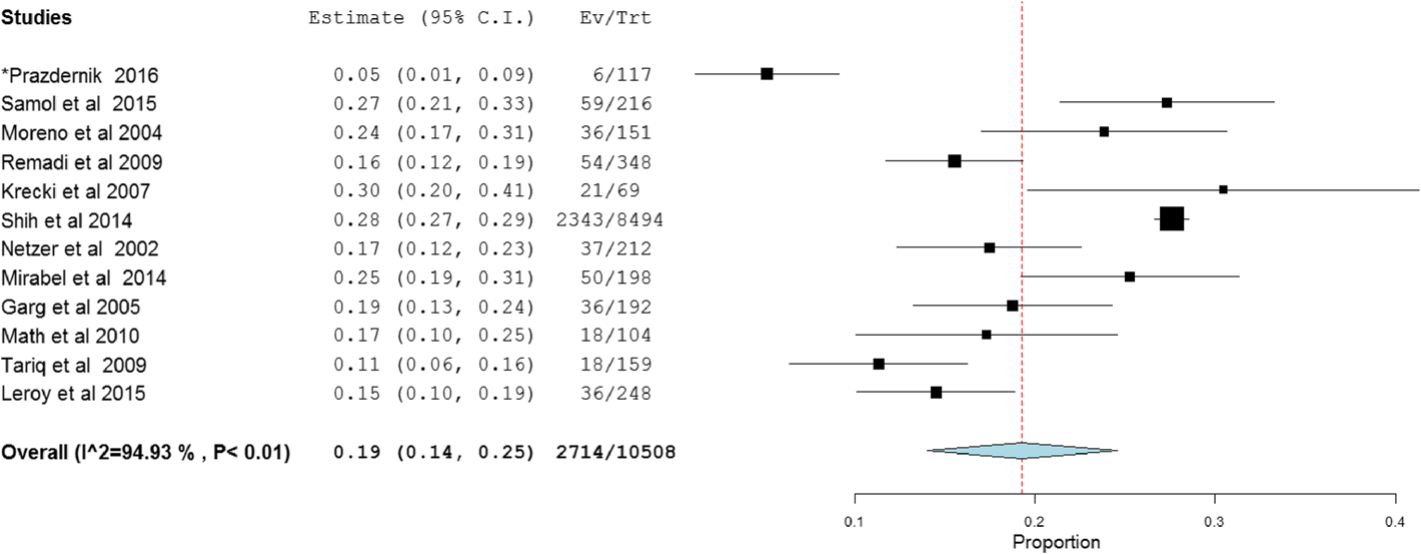
Renal complications of infective endocarditis. *117 episodes of care for 106 patients were occurred and 117 was used as a denominator in the calculation

Complication of IE including renal, cardiac and embolic (septic) were evaluated in 13,637 patients, of which at least one complication was reported in 10,483 (76.9%) patients. The pooled prevalence of cardiac complications in patients with IE was found to be 39% (95%CI: 32.0–46.0, *P* < 0.01; heterogeneity I^2^ = 98.2%) while septic embolism and renal complications of IE accounted for 25% (95% CI: 20.0–31, P < 0.01; I^2^ = 97.1%) and 19% (95% CI: 14.0–25.0, P < 0.01; heterogeneity I^2^ = 94.9%), respectively (Figs. 4, 5 and 6).

#### Sensitivity and subgroup analysis

The sensitivity analysis showed that omission of anyone of the included studies did not affect the pooled results for both primary and secondary outcomes (all *P* < 0.05). We performed subgroup analysis in those studies which clearly reported the types of IEs. Accordingly, the most frequently reported type of IE was native valve, occurred in 74% (95% CI: 64–84) of patients followed by prosthetic valve IE, 19% (95% CI: 14–23). In terms of sex, subgroup analysis showed that a significantly higher IE rate was reported in males than in females (OR: 4.1; 95% CI: 3.38–4.97) (Appendix 1).

#### Publication bias

Funnel plots supplemented by statistical tests confirmed there existed some evidence of publication bias in the mortality outcome collected during the short-term follow up (Egger’s test, *P* = 0.01; Begg’s test, *P* = 0.18), as well as in the long-term (Egger’s test, *P* = 0.03; Begg’s test, *P* = 0.02) (Appendix 2).

## Discussion

To our knowledge, this is the first systematic review and meta-analysis to explore the short-and long-term outcomes in IE patients. In our meta-analysis, a higher proportion of mortality was found in long-term IE patients and the most frequently reported type of IE was associated with native valve involvement. Further analysis suggested that a significant variation in percentage of patients with IE was noticed between the sexes. Despite recent advancement in treatment, IE remain a lethal disease following surgery with long-term (1-year and 5-year) mortality of 40% and 70%, respectively [24]. This is higher in patients when the causative microorganism is *Staphylococcus aureus* that causes serious valvular damage and is also associated with higher embolization and mortality [42]. A fifteen year cohort study has indicated more than 50% death over a follow-up period of 89 months before the year 1995 [27]. Unlike our finding which reported 37% mortality from long-term follow-up, this difference might be due to the variation in the study period in which our included studies were published after 2000, and in fact, many advanced changes in treatment and care of IE patients may likely reduce this occurrence. After short-term survivors of IE, a twenty five years follow-up study indicated a long-term survival rate of less than 50% [43]. Prospective study of non-drug addicts has found a long-term mortality rate of 29% over ten year follow-up [43]. In the present study, native valve involvement was frequently observed. A prospective cohort study from 28 countries indicated that native valve IE was a common scenario both in the community and hospital settings [44]. This may be due to little to no effect of the use of prophylaxis for the prevention of native valve IE during surgical procedures [45]. Evidence obtained from seven electronic databases in five countries indicated that mortality and staphylococcus infections are more prominent in native valve IE [46]. A multicenter cohort study has also revealed that bacterial characteristics may contribute to the occurrence of IE in patients with *Staphylococcus aureus* bacteremia [47]. Multiple studies examined the in-hospital and 30-days mortality in native valve patients ranging from 3.2 to 15.5% [38, 48–51]. Our findings discovered somewhat higher short-term mortality, irrespective of the type of IE. The relatively poor outcomes in short-term may be influenced by multiple factors which include valve characteristics, host factors, causative organisms, development of intra-cardiac, or systemic complications and the therapeutic options. Furthermore, more frequent abscess formation and complete valve damage may be associated with poor outcomes in short- and long term-basis [51]. Although we did not analyze mortality according the pathogen involved in IEs but a previous study [52] demonstrated no difference in mortality between culture negative and culture positive endocarditis. Still, *Staphylococcus aureus* appeared as a leading pathogen, with an overall in-hospital mortality rate of 45% [52]. In addition, large vegetation size and presence of more than one vegetation are associated with higher probability of death [53]. In-hospital mortality could also be higher due to delayed diagnosis and initiation of empiric therapy [54]. Similarly, a previous study [55] revealed a significant difference in mortality between native valve and prosthetic valve endocarditis.

IE is one of the most common and serious complication of intravenous drug use (IVDU) which mainly involve the tricuspid valve and the most isolated etiology being *Staphylococcus aureus,* isolated in 68% of IVDU-IE patients [56, 57]. Through this review, we identified the occurrence of IE in IVDU was 18%. The absolute mortality of IE in IVDU is difficult to find in the literatures. Some studies estimated an in-hospital mortality ranging from 5 to 20% [58, 59]. Another study identified that acute infection accounted for approximately 60% of hospital admission and that IVDU-IE was implicated in 5–15% of these episodes [60]. Predictive IE in IVDU patients includes cocaine use, and signs of septic emboli, cavity, or effusion on chest x-ray [61].

The current study has also indicated that complications of IE were considered to be more prevalent. Particularly, cardiac complications were more prominent than renal and septic/embolic complications. This may be due to the wide variety of manifestations of the cardiac complications including peri-annular abscesses, fistulae, acute coronary syndrome, and pericarditis [62]. But, neurologic complications were not examined in the present study. Therefore, neurologic sequelae of IE is a subject of interest and should be investigated in the future perspective.

Additionally, in our study, the risk of developing IE was higher in males than in females. This is consistent with Levine et al. study [60], and in that study men with IE were older than females (mean age: 32.7 years vs. 31.4 years) and have significantly lower histories of addiction. Previous studies [63, 64] reported no significant differences in in-hospital prognosis and mortality between men and women with left-sided IE. However, still concrete evidence is scarce to support this claim.

### Limitation of the study

The present review disclosed the rate of short- and long-term mortality in IE patients. But, it is not without limitations. Firstly, some of the studies included in the review showed higher level of heterogeneity and we could not detect the source of variation with the available data. While we planned to stratify the primary outcomes into various covariates, it was difficult to extract data in such a way that allowed us to calculate sub-group analysis. Secondly, some evidence of publication bias confirmed in this review, suggesting that many observational studies with rigorous designs are warranted. Also, we urge cautious interpretation of the pooled results given the limitation on our English language search strategy coupled with fewer databases searched. Thirdly, the strict quality assessment score showed that six out of 25 studies were low in quality and were thus, regarded to be at higher risk of bias. In these studies, for instance, sample size calculation was infrequently done and underpowered. However, we conducted a sensitivity analysis to avoid the ‘drowning effect’ from large sample size studies – for example, one-on-one exclusion of Ternhag et al. 2013 [17] and Shih et al. 2014 [33] in the short-term mortality did not differ from the original overall estimates.

## Conclusion

A significantly higher proportion of mortality was found in short- and long-term follow-up of IE patients and the most frequently reported type of IE was associated with native valve involvement. The burden of IE complications were higher among IE patients and were mostly cardiovascular. In addition, a significantly higher IE rate was reported in males than in females. Further research is needed to assess the determinants of overall mortality in IE patients, as well as well-designed observational studies to conform our results.

## Figures and Tables

**Fig. 7:**
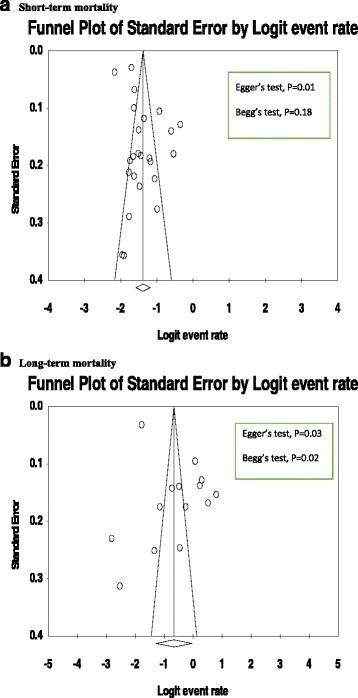
Sub-group analysis. **a**. Types of IE. **b**. Sex

**Fig. 8:**
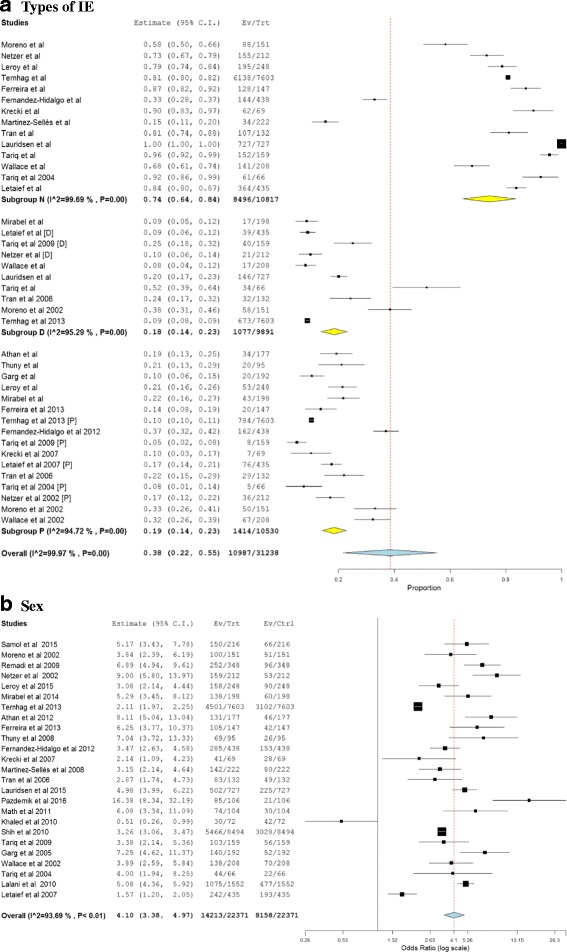
Publication bias. **a**. Short-term mortality. **b**. Long-term mortality

## Additional file

Additional file 1: Final excluded studies with reasons (1). Studies excluded from the systematic review and meta-analysis after full text (*N* = 26). This file contains list of studies that have been excluded during the literature review process due to lack of fulfilling the inclusion criteria into the systematic review and meta-analysis. (PDF 448 kb)

## Abbreviations

BCNE: Blood culture negative endocarditis
BCPE: Blood culture positive endocarditis
CHD: Congenital heart disease
GBD: Global burden of disease
HACEK: Haemophilus, Aggregatibacter, Cardiobacterium hominis, Eikenella corrodens, Kingella species
HIV: Human immune virus
IE: Infective endocarditis
IV: Intravenous
IVDU: Intravenous drug users
OR: Odds ratio
RHD: Rheumatic heart disease
STROBE: Strengthening the Reporting of Observational Studies in Epidemiology
