# Correction to: Short- and long-term outcomes in infective endocarditis patients: a systematic review and meta-analysis

**DOI:** 10.1186/s12872-018-0742-3

**Published:** 2018-01-12

**Authors:** Tadesse Melaku Abegaz, Akshaya Srikanth Bhagavathula, Eyob Alemayehu Gebreyohannes, Alemayehu B. Mekonnen, Tamrat Befekadu Abebe

**Affiliations:** 1 Department of Clinical pharmacy, School of Phamacy, College of Medicine and Health Sciences, University of Gondar, Gondar, Ethiopia; 2 Medication Safety Chair, College of Pharmacy, King Saud University, Riadh, Saudi Arabia; 3 Master’s Program in Health Economics, Policy and Managment; Student; Department of Learning, Informatics, Managent and Ethics, Karolinska Institutet, Solna, Sweden

## Correction

Unfortunately, after publication of this article [1], it was noticed that the name of the second author was incorrectly displayed as Akshaya Srikanth Bahagavathula. The correct name is Akshaya Srikanth Bhagavathula and can be seen in the corrected author list above. The original article has also been updated to correct this error.
