# Supplementary material for: Short- and long-term outcomes in infective endocarditis patients: a systematic review and meta-analysis
Source: BMC Cardiovasc Disord. 2017 Dec 12;17:291. doi: 10.1186/s12872-017-0729-5 (PMC5728061; doi:10.1186/s12872-017-0729-5)
Supplement: Additional file 1: — Final excluded studies with reasons (1). Studies excluded from the systematic review and meta-analysis after full text (N = 26). This file contains list of studies that have been excluded during the literature review process due to lack of fulfilling the inclusion criteria into the systematic review and meta-analysis. (PDF 448 kb) [file 12872_2017_729_MOESM1_ESM.pdf]

Studies excluded from the systematic review and meta-analysis after full text (N=26).

s.no	Title	Reason
**1**	Kim JB, Ejiofor JI, Yammine M, Ando M, Camuso JM, Youngster I, Nelson SB, Kim AY, Melnitchouk SI, Rawn JD, MacGillivray TE. Surgical outcomes of infective endocarditis among intravenous drug users. The Journal of thoracic and cardiovascular surgery. 2016 Sep 30;152(3):832-41.	Focused on outcomes of surgical management.
2	Kamaledeen A, Young C, Attia RQ. What are the differences in outcomes between right-sided active infective endocarditis with and without left-sided infection?. Interactive cardiovascular and thoracic surgery. 2011 Nov 30:ivr012.	It is review paper focused on outcomes of surgical interventions.
3	Alexiou C, Langley SM, Monro JL. Surgery for infective valve endocarditis in children. European journal of cardio-thoracic surgery. 1999 Dec 1;16(6):653-9.	It focused on outcomes of surgical interventions in children
4	Roca B, Marco JM. Presentation and outcome of infective endocarditis in Spain: a retrospective study. International journal of infectious diseases. 2007 May 31;11(3):198-203.	It didn't specify short term or long term outcomes
5	Cicalini S, Puro V, Angeletti C, Chinello P, Macrì G, Petrosillo N. Profile of infective endocarditis in a referral hospital over the last 24 years. Journal of Infection. 2006 Feb 28;52(2):140-6.	It didn't state the level of outcome
6	Kaen K. Effect of changes in diagnosis and management of active infective endocarditis on the clinical outcome at Srinagarind Hospital. J Med Assoc Thai. 2005;88(4):498-504.	It didn't clearly state the overall long term outcome of treatment among the study subjects
7	Carozza A, De Santo LS, Romano G, Corte AD, Ursomando F, Scardone M, Caianiello G, Cotrufo M. Infective endocarditis in intravenous drug abusers: patterns of presentation and long-term outcomes of surgical treatment. Journal Of Heart Valve Disease. 2006 Jan 1;15(1):125.	It measured surgical treatment outcome
8	Perez de Isla L, Zamorano J, Lennie V, Vázquez J, Ribera JM, Macaya C. Negative blood culture infective endocarditis in the elderly: long-term follow-up. Gerontology. 2007 Apr 11;53(5):245-9.	It didn't measure primary and secondary outcomes
9	Castillo JC, Anguita MP, Ramirez A, Siles JR, Torres F, Mesa D, Franco M, Munoz I, Concha M, Valles F. Long term outcome of infective endocarditis in patients who were not drug addicts: a 10 year study. Heart. 2000 May 1;83(5):525-30.	It included children.
10	Tornos-Mas MP, Permanyer-Miralda G, Planes-Reig A, Serrat-Serradell R, Del Castillo HG, Soler-Soler J. Long-term follow-up of native valve infective endocarditis. European heart journal. 1984 Oct 1;5(suppl C):107-10.	It included children.
11	Danchin N, Retournay G, Stchepinsky O, Selton-Suty C, Voiriot P, Hoen B, Canton P, Villemot JP, Mathieu P, Cherrier F. Comparison of long term outcome in patients with or without aortic ring abscess treated surgically for aortic valve infective endocarditis. Heart. 1999 Feb 1;81(2):177-81.	It measured surgical treatment outcome
12	Mueller XM, Tevaearai HT, Stumpe F, Fischer AP, Hurni M, Ruchat P, von Segesser LK. Multivalvular surgery for infective endocarditis. Vascular. 1999 Jun 1;7(4):402-8.	It measured surgical treatment outcome
13	Hricak V, Kovacik J, Marks P, West D, Kromery V. Aetiology and outcome in 53 cases of native valve staphylococcal endocarditis. Postgraduate medical journal. 1999 Sep 1;75(887):540-3.	Low quality, didn't state male: female ratio, didn't investigate acute phase of IE.
15	Prendergast BD, Tornos P. Surgery for Infective Endocarditis Who and When?. Circulation. 2010 Mar 9;121(9):1141-52.	It measured surgical treatment outcome
16	Mccarthy JT, Steckelberg JM. Infective endocarditis in patients receiving long-term hemodialysis. Mayo Clinic Proceedings. 2000 Oct 31; 75(10):1008-1014. Elsevier.	It focused only on incidence of IE in patients on hemodialysis

17	Renzulli A, Carozza A, Marra C, Romano G, Ismeno G, De Feo M, Della Corte A, Cotrufo M. Are blood and valve cultures predictive for long-term outcome following surgery for infective endocarditis?. European journal of cardio-thoracic surgery. 2000 Mar 1;17(3):228-33.	It focused only on impact of bacteria on surgical outcome
18	Moura L, Zamorano J, Moreno R, Almeria C, Rodrigo JL, Villate A, Móran J, Sanchez-Harguindey L. Perioperative mortality and long-term outcome of infective endocarditis. Portuguese journal of cardiology. 2002 Sep;21(9):989-99.	It measured surgical treatment outcome
19	Pompilio G, Brockmann C, Bruneau M, Buche M, Amrani M, Louagie Y, Eucher P, Rubay J, Jamart J, Dion R, Schoevaerdts JC. Long-term survival after aortic valve replacement for native active infective endocarditis. Vascular. 1998 Apr 1;6(2):126-32.	It measured surgical treatment outcome
20	Spiliopoulos K, Giamouzis G, Haschemi A, Karangelis D, Antonopoulos N, Fink G, Kemkes BM, Gansera B. Surgical Management of Infective Endocarditis: Early and Long-Term Mortality Analysis. Single-Center Experience and Brief Literature Review. Hellenic J Cardiol. 2014 Nov 1;55:462-74.	It measured surgical treatment outcome
21	Sadiq M, Nazir M, Sheikh SA. Infective endocarditis in children—incidence, pattern, diagnosis and management in a developing country. International Journal of cardiology. 2001 Apr 30;78(2):175-82.	The study participants were children. It measured surgical treatment outcome.
22	Rabkin DG, Mokadam NA, Miller DW, Goetz RR, Verrier ED, Aldea GS. Long-term outcome for the surgical treatment of infective endocarditis with a focus on intravenous drug users. The Annals of thoracic surgery. 2012 Jan 31;93(1):51-7.	It measured surgical treatment outcome
23	Tom KaiMingWang et al Characteristics and Outcomes for Right Heart Endocarditis: Heart, Lung and Circulation: 2014,	Small sample size
24	Sun XL, Zhang J, Wang GG, Zhuang XF, Yang YM, Zhu J, Tan HQ, Yu LT. Comparison of characteristics and short-term outcome from fungal infective endocarditis in prosthetic valve endocarditis versus native valve endocarditis. The American journal of cardiology. 2013 Jul 1;112(1):111-6	Small sample size
25	Mirabel M, A, et al. Infective endocarditis in the Pacific: clinical characteristics, treatment and long-term outcomes. open heart. 2015	Small sample size
26	Chan KL. Early clinical course and long-term outcome of patients with infective endocarditis complicated by perivalvular abscess. Canadian Medical Association Journal. 2002 Jul 9;167(1):19-24.	Small sample size
